# 4-Phenylbut-2-yl Esters from the Essential Oil of *Artemisia rutifolia* from Mongolia

**DOI:** 10.3390/molecules31060926

**Published:** 2026-03-11

**Authors:** Elisa Irrera, Yea Jee Ahn, Shatar Sandui, Altantsetseg Shatar, Nicolas Baldovini

**Affiliations:** 1 Institut de Chimie de Nice, UMR 7272, Université Côte d’Azur, Parc Valrose, 06108 Nice, France; elisa.irrera@studenti.unime.it (E.I.); yeajeeahn@gmail.com (Y.J.A.); 2 Messina Institute of Technology c/o Department of Chemical, Biological, Pharmaceutical and Environmental Sciences, University of Messina, Viale G. Palatucci 13, 98168 Messina, Italy; 3 Institute of Chemistry and Chemical Technology, Mongolian Academy of Sciences (MAS), Ulaan-Baatar 211051, Mongoliaaltaa12000@yahoo.com (A.S.)

**Keywords:** *Artemisia*, *Artemisia rutifolia*, 4-phenylbut-2-yl esters, phenylbutanoids, essential oils

## Abstract

Most species belonging to the genus *Artemisia* are aromatic plants showing a broad diversity in their essential oil composition. *Artemisia rutifolia*, traditionally used in folk medicine, exhibits an atypical chemotype characterized by a high concentration of phenylbutanoids, in contrast to the profiles observed in other specimens of the same species. This study aimed to provide an in-depth chemical characterization of the phenylbutanoid-rich essential oil of *A. rutifolia* obtained from samples collected in the Middle Gobi province of Mongolia. Particular attention was devoted to the identification of the minor phenylbutanoids and a preliminary determination of the main contributors to the odor of the oil. Hence, the essential oil was fractionated by column chromatography and subjected to GC-MS/FID and GC-O/FID analyses. Camphor, 1,8-cineole, and 4-phenylbutan-2-one were identified as the dominant compounds, the latter being the main odorant responsible for the typical fresh-fruity smell of the plant. Moreover, α- and β-thujones were absent, and seven previously unreported 4-phenylbut-2-yl esters were unambiguously identified through combinatorial synthesis. These findings highlight the chemical distinctiveness of the Middle Gobi chemotype and support its potential for industrial essential oil production due to its high yield, lack of thujones, and pleasant fresh aroma.

## 1. Introduction

The genus *Artemisia* (Asteraceae) comprises approximately 500 species widely distributed throughout the Northern Hemisphere, mostly in Central Asia, East Asia, Europe, and North America, as well as in arid and semi-arid regions of North Africa [1]. Many of these species are medicinal plants, among which one of the most famous is *A. annua*, a source of the antimalarial drug artemisinin [2]. Other important species include *A. vulgaris* (mugwort), a common plant with a long history of use in folk medicine across Europe and Asia, and *A. absinthium* (wormwood), well known as the key ingredient of the alcoholic beverage absinthe [3]. Most of the *Artemisia* species are aromatic plants, with a broad diversity of essential oil compositions, often displaying several chemotypes within the same species [4]. *Artemisia* essential oils are generally characterized by a high content of common monoterpenoids (camphor, 1,8-cineole, pinenes…), a frequent occurrence of α- and β-thujones, and the occasional presence of some less classical constituents like the irregular terpenoids artemisia ketone and davanone. Much less frequently, non-terpenic volatiles have also been reported within this genus, like the phenylpropanoids methyl chavicol, methyl eugenol, and elemicin in *A. dracunculus* [5]. From the point of view of the biosynthetic origin of its volatiles, one of the most atypical *Artemisia* species is *A. rutifolia* Steph. ex Spreng. occurring in mountain steppes and rocky slopes across Central and Inner Asia. It is a perennial semi-shrub with woody and branched stems that can reach up to 80 cm in height. Its stems and divided leaves are glabrescent and silky puberulent with a silvery-gray appearance [6,7]. It has been used as a tonic, febrifuge, and anthelmintic in folk medicine [8].

The first report on the composition of the essential oil of *A. rutifolia* mentioned rather classical constituents (1,8-cineole, camphor, thujones) in samples collected in Tajikistan [9] but later, Shavarda used a combination of preparative GC, NMR, IR and synthetic transformations to show that plants growing in Tuva republic (Siberia) contained significant amounts of racemic 4-phenylbutan-2-ol **1**, accompanied by its corresponding acetate **2** and ketone (4-phenylbutan-2-one) **3** [10]. The presence of these uncommon aromatic compounds in *A. rutifolia* was confirmed later, with **3** being even the major constituent of samples from Middle Gobi (Mongolia) [11]. However, **1**–**3** could not be identified, even in trace amounts, in the plants growing wild in the Pamir mountains of Tajikistan, which contained instead mostly α- and β-thujones [12]. Recently, Dylenova et al. [13] concluded that *A. rutifolia* exists as two distinct chemotypes with a clear geographical distribution: plants growing in Buryatia and Mongolia characterized by high contents of **3** and camphor, and plants from Tajikistan containing mainly α- and β-thujones. Nevertheless, the chemotaxonomy of *A. rutifolia* still needs further investigations on a larger set of samples, since the occurrence of phenylbutanoids is not systematic in samples collected in Mongolia. Indeed, compositions dominated either by santolinatriene and myrcene, or camphor, cineole, and carvacryl methyl ether were also reported for plants originating from Uvs province and the Selenga river area, respectively [14,15].

In contrast with the naturally widespread compounds of *Artemisia* species, the non-terpenic constituents **1**–**3** are much less common in natural sources, and to the best of our knowledge, have never been reported in other *Artemisia* species so far. The biosynthetic origin of these compounds is also puzzling, since their four-carbon chain implies that they cannot be classified as phenylpropanoids, and the metabolic pathways producing them have not yet been investigated up to now. In this context, we were interested in better characterizing the composition of *A. rutifolia* essential oil containing such phenylbutanoids. While previous studies have focused exclusively on the major components **1**–**3**, we hypothesized that other minor constituents, particularly 4-phenylbut-2-yl esters, might be present in the oil. Therefore, we performed a detailed analysis of the essential oil of samples collected in Middle Gobi, Mongolia, using combinatorial synthesis to identify these undocumented minor constituents, and characterizing the main odorant compounds through GC-O analysis.

## 2. Results

**Figure 1:**
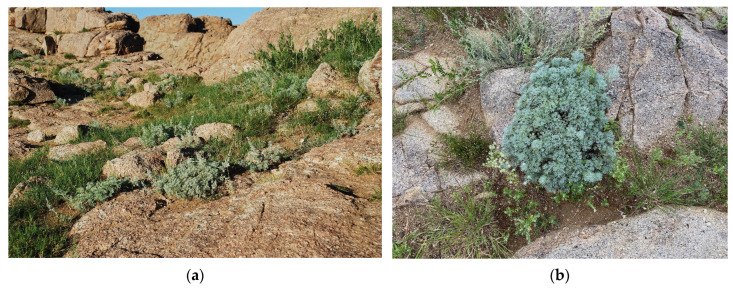
(**a**) Plant material collection site in Baga gazariin chuluu, Middle Gobi, Mongolia. (**b**) *Artemisia rutifolia* specimen.

In order to prepare an essential oil from the phenylbutanoid-rich chemotype of *A. rutifolia*, the vegetal material was collected in Middle Gobi Province, where the presence of this chemotype has already been reported [11] (Figure 1). Table 1 describes the composition of this essential oil, analyzed by GC-MS/FID with two columns of different polarities. To improve the reliability of the analyses, remove some coelutions and characterize additional trace constituents, the essential oil was subjected to fractionation by column chromatography on silica gel. Elution was performed using 10 mL portions of solvent mixtures with progressively increasing polarity, affording sequentially seven fractions. Each fraction was subsequently analyzed under the same GC conditions as the whole oil and used to support and refine compound identification. The composition of the oil was consistent with that reported by Trendafilova et al. [11]. Camphor, 1,8-cineole, and **3** were the main constituents of the oil, which also contained **1**–**2** in addition to classical mono- and sesquiterpenic compounds, as well as some phenylpropanoids. The complete absence of α- and β-thujones is noteworthy, like the presence of trace amounts of chamazulene in the first fraction, which possessed a typical blue color and was responsible for the green color of the total oil. The presence of this compound is probably related to the occurrence of hydroxylated guaianolides in *A. rutifolia* [16,17].

**Scheme 1:**
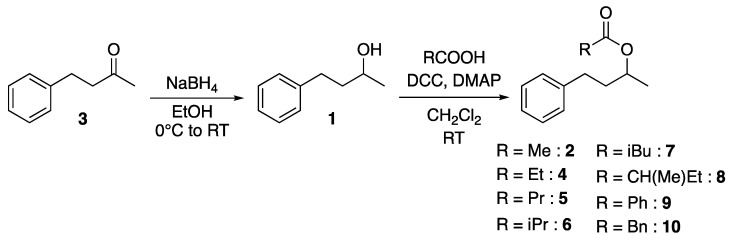
Synthesis of 4-phenylbut-2-yl esters and structures of *A. rutifolia* constituents already reported (**1**–**3**) [10] and identified in this study (**4**–**10**).

**Figure 2:**
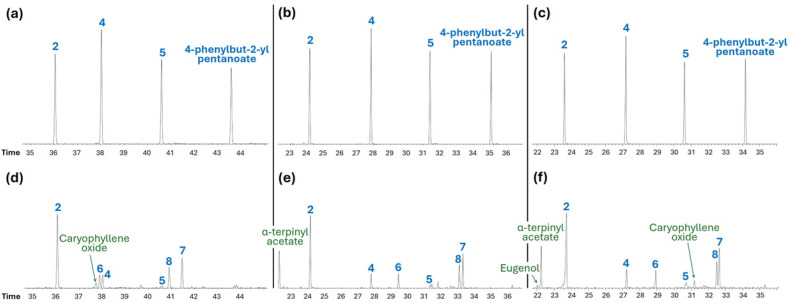
GC-MS chromatograms of the combinatorial mixture of 4-phenylbut-2-yl *n*-alkanoates (top) (**a**–**c**) and of the third fraction of *A. rutifolia* essential oil (bottom) (**d**–**f**), analyzed by DB-WAX (**a**,**d**), SLB-5ms (**b**,**e**), and DB-1ms (**c**,**f**) columns.

A closer look at the ester-rich third chromatographic fraction (eluted with petroleum ether/diethyl ether 95/5) helped us to locate interesting constituents whose MS spectra were not present in our database. We suspected that these compounds might be 4-phenylbut-2-yl esters, homologues of **2**, as their retention indices were higher, and their mass spectra showed characteristic abundant fragments observed for **2**, at *m*/*z* = 91, 117, and 132. Because of the low amount of the fraction of interest, any attempt to characterize these compounds by the classical fractionation approach was hopeless, so we opted for the use of combinatorial synthesis to quickly screen for the presence of 4-phenylbut-2-yl esters in the fractions and confirm their presence in the oil unambiguously. This approach is a potent method that has been used successfully for the identification of new esters [18], ethers [19], and ketones [20] in essential oils. In this work, we first used the Steglich procedure (Scheme 1) [21] to esterify 4-phenylbutan-2-ol **1** with acetic acid to prepare a standard of pure ester **2** for the confirmation of its presence in the oil, and also to ensure that the esterification reaction was clean enough to be adapted to the constraints of the combinatorial synthesis of reference compounds for GC analyses (i.e., total conversion of the reagents and clean crude products) [22]. Then, this reaction could be applied to an equimassic mixture of *n*-alkanoic acids (from acetic acid to palmitic acid), and the GC-MS chromatograms (obtained on three different columns) of the resulting ester mixture were compared to those of the third fraction of *A. rutifolia* essential oil. Hence, we could confirm the presence of 4-phenylbut-2-yl propionate **4** and 4-phenylbut-2-yl butyrate **5** (Figure 2).

Several additional constituents showing a rather similar MS spectrum eluted at a RI_DB-1ms_ consistent with the one expected for 4-phenylbut-2-yl esters derived from four and five-carbon acids. Therefore, we also synthesized 4-phenylbut-2-yl isobutyrate **6** and isovalerate **7** (as a combinatorial mixture), as well as 2-methylbutyrate **8** (as a single compound), following the same procedure. By comparing the MS and RI data of these esters on the three GC columns with those of the third fraction of the oil, we could unambiguously demonstrate the presence of **6**–**8** in *A. rutifolia* essential oil. Finally, two constituents displaying also the characteristic MS fragmentation of 4-phenylbut-2-yl esters eluted at RI_DB-1ms_ = 1930 and 1978, which did not fit with any of the RI_DB-1ms_ observed for the *n*-alkanoates. Moreover, their MS spectrum did not contain the expected fragments of the alkanoyl moiety, but on the contrary showed increased abundance of the fragment at *m*/*z* = 91, as well as additional fragments at *m*/*z* = 77 and 105 for the compound at RI_DB-1ms_ = 1930. Therefore, we suspected that it could be an aryl ester of **2**, and repeated the combinatorial synthesis with a mixture of benzoic, phenylacetic, and phenylpropionic acids. As a result, the RI and MS data of 4-phenylbut-2-yl benzoate **9** and phenylacetate **10** were in agreement with those of the two initially unidentified esters, and showed that **9**–**10** were also constituents of the oil.

**Table 1:** Chemical composition of the *A. rutifolia* essential oil.

RI_DB-1ms_^a^	RI_DB-Wax_^b^	Compound	% ^c^	Identification ^d^
925	922	benzaldehyde	0.03	Std
928		α-pinene	0.60	Std
940	482	camphene	0.52	Std
963	530	sabinene	0.14	MS, RI
967	516	β-pinene	0.12	Std
976	599	2,3-dehydro-1,8-cineole	0.15	MS
1008	590	α-terpinene	0.31	MS, RI
1010	680	para-cymene	0.20	Std
1018	617	1,8-cineole	9.21	Std
1020	609	limonene	0.19	Std
1030	1043	acetophenone	0.03	Std
1046	656	γ-terpinene	0.67	Std
1049	871	*cis*-sabinene hydrate	0.58	MS, RI
1076	693	terpinolene	0.15	MS, RI
1079	950	*trans*-sabinene hydrate	0.61	MS, RI
1080	841	filifolone	tr	MS
1096	905	chrysanthenone	0.43	MS, RI
1102	952	4-acetyl-1-methylcyclohexene	0.20	MS, RI
1117	913	camphor	11.14	Std
1133	962	pinocarvone	0.14	MS, RI
1143	1147	*cis*-chrysanthenol	0.82	MS, RI
1157	1003	terpinen-4-ol	2.11	Std
1168	1095	α-terpineol	2.02	Std
1172	1067	estragole	tr	Std
1177	1184	myrtenol	tr	MS, RI
1197	1428	3-phenylpropan-1-ol	tr	Std
1209	1251	4-phenylbutan-2-one **3**	40.02	Std
1227	1380	4-phenylbutan-2-ol **1**	6.41	Std
1236	1125	benzyl acetate	tr	Std
1242	971	*cis*-chrysanthenyl acetate	0.08	MS, RI
1249	1803	indole	tr	Std
1265	979	bornyl acetate	0.04	Std
1280	1015	terpin-1-en-4-yl acetate	0.03	MS, RI
1313	1488	4-phenyl-3-buten-2-one	0.11	Std
1314	1132	*trans*-carvylacetate	tr	MS, RI
1323	1544	eugenol	0.37	Std
1328	1092	α-terpinyl acetate	0.32	MS, RI
1330	885	bicycloelemene	0.06	MS, RI
1360	1322	*cis*-jasmone	0.22	MS, RI
1363	1305	4-phenylbut-2-yl acetate **2**	0.79	Std
1368	1400	methyleugenol	tr	Std
1369	894	α-copaene	0.12	Std
1408	993	(*E*)-β-caryophyllene	0.21	Std
1430	1001	aromadendrene	tr	MS, RI
1442	1061	α-humulene	tr	Std
1449	1038	allo-aromadendrene	tr	MS, RI
1450	1655	4-(4-methoxyphenyl)butan-2-one	0.45	Std
1453	1364	4-phenylbut-2-yl propionate **4**	0.13	Std
1467	1100	germacrene D	0.21	MS, RI
1471	1775	4-(4-methoxyphenyl)-butan-2-ol	tr	MS
1482	1125	bicyclogermacrene	0.94	MS, RI
1497	1360	4-phenylbut-2-yl isobutyrate **6**	0.17	Std
1499	1150	γ-cadinene	tr	MS, RI
1508	1151	δ-cadinene	0.03	MS, RI
1520	1298	α-calacorene	tr	MS, RI
1544	1442	4-phenylbut-2-yl butyrate **5**	tr	Std
1552	1497	spathulenol	0.17	MS, RI
1557	1355	caryophyllene oxide	0.12	Std
1562	1450	globulol	0.16	MS, RI
1581	1550	copaborneol	0.27	MS, RI
1592	1452	4-phenylbut-2-yl 2-methylbutyrate **8**	0.16	Std
1595	1469	4-phenylbut-2-yl isovalerate **7**	0.23	Std
1601	1699	methyl jasmonate	0.11	MS, RI
1615	1545	τ-cadinol	0.41	MS, RI
1627	1601	α-cadinol	0.09	MS, RI
1659	1592	α-bisabolol	0.23	MS, RI
1672		4-phenylbut-2-yl alkanoate ^e^	tr	
1692	1746	chamazulene	tr	MS
1702		4-phenylbut-2-yl alkanoate ^e^	tr	
1714		benzyl benzoate	tr	MS, RI
1801		phenylethyl benzoate	tr	MS
1898		unknown ^f^	0.66	-
1930		4-phenylbut-2-yl benzoate **9**	tr	Std
1943		hexadecanoic acid	tr	Std
1978		4-phenylbut-2-yl phenylacetate **10**	0.17	Std

^a^ RI_DB-1ms_ = linear retention indices determined in relation to a homologous series of *n*-alkanes (C_8_-C_40_) on an DB-1ms column; ^b^ RI_DB-Wax_ = linear retention indices determined from the retention times of a series of methyl *n*-alkanoates with linear interpolation (RI = 100 × number of carbons of the acid moiety); ^c^ Mass percentage determined by GC-FID using predicted response factors [23], with tetradecane as an internal reference. tr = trace (< 0.01%); ^d^ Identification method: MS = constituent identified by comparison of its mass spectrum with those listed in the literature or in a homemade mass spectral library built with authentic reference compounds; RI = constituent identified by comparison of its retention index with those listed in the literature or in a homemade retention index library built with authentic reference compounds; Std = identification confirmed by injection of an authentic reference compound. ^e^ Alkanoate moiety is not identified. ^f^ MS (EI, 70 eV), *m*/*z* (rel. int.): 230 (69), 215 (24), 185 (13), 169 (16), 159 (35), 145 (34), 133 (81), 119 (46), 105 (100), 91 (79), 77 (50), 53 (52).

## 3. Discussion

The essential oil of *Artemisia rutifolia* examined in this study showed a phenylbutanoid profile dominated by 4-phenylbutan-2-one and **3** and 4-phenylbutan-2-ol derivatives, consistent with earlier studies on this specific chemotype [10,11,13]. While **1**–**3** have been previously reported in this species and in other natural sources, the esters **4**–**10** have not been documented as natural products up to now. Overall, the results highlight *A. rutifolia* as a chemically distinctive taxon within the genus and underscore the importance of regional chemotype investigations in revealing hidden natural product diversity.

In view of the pleasant specific odor of the plant, we were also interested in investigating the identity of the main olfactory contributors of its essential oil with the help of GC-O. The alcohol **1** and its esters **2**, **4**–**10** exhibit a very weak odor and therefore contribute only marginally to the overall aroma profile of the plant. In contrast, GC–O analysis (3 panelists) identified **3**, which constitutes approximately 40% of the essential oil, as a key contributor to the characteristic fresh-fruity odor of the plant. This compound, described by the panelists as fruity, herbal, sweet, warm, and slightly woody, provides a warm and soft heart note in aromatic matrices. It has also been reported as an important contributor to the top note of agarwood oil and burning oud smoke, and has been associated with mild sedative effects [24]. In addition, the monoterpenoids 1,8-cineole and camphor represent additional major constituents of this oil, imparting fresh, minty, balsamic, and herbal top notes to the essential oil. Eugenol imparts a clove-like odor characterized by warm, spicy, and subtly sweet notes. Even if its amount in the oil is not very high, its olfactory impact is probably significant in view of its low odor threshold, reinforcing the warm heart-note characteristics of the oil. Overall, the aroma profile results from the synergistic interaction between fresh and cooling monoterpenoids (1,8-cineole and camphor), warm notes of eugenol, and sweet–balsamic facets of **3**, which together shape the characteristic olfactory signature of the plant. In conclusion, the Middle Gobi chemotype of *Artemisia rutifolia* represents a promising species for industrial essential oil production due to its high yield, widespread availability in the wild, absence of thujones, and a distinctive, pleasant aroma with agarwood-like top note.

## 4. Materials and Methods

### 4.1. General Procedures

Chemicals for synthesis and fractionation were purchased from Sigma–Aldrich (St. Louis, MO, USA), Macherey-Nagel (Düren, Germany), and VWR International (Radnor, PA, USA). All solvents (petroleum ether (PE), methyltertbutylether (MTBE) (purity ≥ 99.8%), diethyl ether (Et_2_O) (purity ≥ 99.8%), dichloromethane (purity ≥ 99.9%) were used as purchased. NMR Analyses were performed on Bruker NanoBay Avance IIIHD (400 MHz) spectrometer and Bruker Avance DRX 500 (500 MHz) spectrometers (Bruker, FR-Wissembourg, France) at 25 °C in CDCl_3_.

### 4.2. Plant Material and Extraction Procedure

The *Artemisia rutifolia* aerial part samples were collected from several individual plants in Baga Gazariin Chuluu, Dundgobi province, Mongolia (46.224° N, 106.002° E (WGS84) in August 2023. A voucher specimen with the reference NB230829a was deposited in the herbarium of the Institute ICN, Nice. The vegetal material (37 g) was hydrodistilled in a glass Clevenger apparatus for 9 h, and the essential oil was obtained as a green oil with a specific fresh-minty odor (530 mg, 1.4%).

### 4.3. Essential Oil Fractionation

The essential oil (305 mg) was fractionated by column chromatography on 3 g silica gel (15–40 μm), using 10 mL portions of a gradient of petroleum ether (PE) and diethyl ether (Et_2_O) mixture to isolate the following fractions (Eluent composition/mass of isolated fraction): (1) PE/13.7 mg, (2) PE/Et_2_O 99/1/0.6 mg, (3) PE/Et_2_O 95/5/27.2 mg, (4) PE/Et_2_O 9/1/111.9 mg, (5) PE/Et_2_O 8/2/66.7 mg, (6) PE/Et_2_O 1/1/38 mg, (7) Et_2_O/16 mg.

### 4.4. GC-MS/FID Analyses

Gas chromatography—Mass spectrometry (GC-MS) analyses were carried out using an Agilent (Santa Clara, CA, USA) 6890N gas chromatograph equipped with a fused silica capillary column DB-1ms (30 m × 0.25 mm i.d., film thickness: 0.25 µm, J&W122-0132). The analytical parameters were the following: the carrier gas was helium at a flow rate of 2 mL/min, the oven temperature was programmed from 60 to 280 °C at 3 °C/min, and completed by a post-run (300 °C, 5 min). Shortened temperature programs were used when possible, keeping the same rate of 3 °C/min. Samples (1 μL of a ca. 10% solution in MTBE) were injected in split mode (ratio varying in function of the sample, from 1/200 for pure compounds to 1/50 for mixtures) and the injector temperature was 250 °C. The column flow was split using a G3184-60065 splitter (Agilent), and transferred to a FID detector (250 °C, hydrogen and air flows at 40 and 450 mL/min., respectively) and to an Agilent 5973N mass selective detector working in electron impact (EI) mode at 70 eV (scanning over 40–450 amu range in SCAN mode). The temperatures of the MS ion source and transfer line were 230 and 280 °C, respectively. Linear retention indices (LRI) were determined from the retention times of a series of *n*-alkanes with linear interpolation. The samples were analyzed as ca. 10% solutions in MTBE or dichloromethane. The main constituents were identified by comparison of their mass spectra and LRI with those of pure compounds registered in commercial libraries and literature data, and with a laboratory-made database built from authentic compounds, with the help of SearchReview software (Version 3.8.0.0, N. Waleson, Leoson, 2024). GC-MS analyses were also performed on two other apparatus equipped with different columns: (a) a column coated with a polar stationary phase, in the same conditions as described above, except for the following parameters: the column was a fused silica capillary column DB-WAX (30 m × 0.25 mm i.d., film thickness: 0.25 µm, J&W). Oven temperature programmed from 40 to 230 °C at 3 °C/min and held isothermal for 20 min. LRI Linear Retention indices (LRI) were determined from the retention times of a series of methyl *n*-alkanoates with linear interpolation (RI = 100 × number of carbons of the acid moiety) (b) a column coated with (5–phenyl)-methylpolysiloxane, in the same conditions as described for the DB-1ms column, except for the following parameters: the column was a fused silica capillary column SLB-5ms (30 m × 0.25 mm i.d., film thickness: 0.25 µm, J&W). Oven temperature programmed from 60 to 246 °C at 3 °C/min and held isothermal for 60 min.

### 4.5. GC-O/FID Analyses

Gas Chromatography-Olfactometry (GC-O/FID) analyses were performed on a Shimadzu (Tokyo, Japan) GC-2010 Gas Chromatograph equipped with a HP-5 capillary column (50 m × 0.32 mm i.d.; 0.53 μm film thickness, J&W), a FID detector, and an ATAS olfactory port OP275 (GL Sciences, Tokyo, Japan) mounted with a glass nasal cone. Samples were analyzed under the following conditions: injection volume: 1.0 μL, in splitless mode. Injector temperature: 250 °C. Oven temperature program: 60 °C to 250 °C at 6 °C/min, then isothermal for 15 min. Carrier gas (nitrogen) flow: 1.50 mL/min. 60% of the flow was directed to the FID while 40% was directed into the heated sniffing port. FID temperature: 250 °C. The oil was injected as a 10% solution. Three evaluators participated in this study and were asked to memorize the odor of the essential oil studied in this work prior to GC-O experiments. To confirm the attribution of the odor zones of the main odorant contributors to their corresponding odorant constituents (namely, camphor, 1,8-cineole, and **3**), injection of pure standards under the same conditions and at the same concentration was performed.

### 4.6. Syntheses of 4-Phenylbutan-2-ol 1 and Its Esters **2**, **4**–**10**

3.87 g (26 mmoles, 1 eq.) of 4-phenylbutan-2-one (**3**) was added to a solution of sodium borohydride (2.1 g, 55 mmoles, 2.1 eq.) in 30 mL of absolute ethanol cooled in an ice bath, and the mixture was stirred for 7 h and allowed to come back to room temperature. The reaction mixture was then cooled again to 0 °C, 2 mL of water was added, and the reaction mixture was stirred overnight. The excess of ethanol was then evaporated on a rotatory evaporator, and the mixture was extracted twice with diethyl ether (25 mL). The organic phases were then gathered, washed successively with 1N hydrochloric acid, saturated sodium hydrogen carbonate solution, and brine. After drying on anhydrous magnesium sulfate, the solvent was evaporated to give 3.69 g (94%) of a pure sample of 4-phenylbutan-2-ol (**1**) used without purification in the next steps. 4-phenylbutan-2-ol **1**: colorless oil, ^1^H NMR (500 MHz, CDCl_3_) δ 7.30–7.24 (m, 2H), 7.21–7.14 (m, 3H), 3.86–3.76 (sext., *J* = 6.1 Hz, 1H), 2.8–2.6 (m, 2H), 1.84–1.66 (m, 2H), 1.23–1.19 (d, *J* = 6.2 Hz, 3H); ^13^C NMR (126 MHz, CDCl_3_) δ 142.18 (C), 128.49 (CH), 125.90 (CH), 67.54 (CH), 40.93 (CH_2_), 32.23 (CH_2_), 23.68 (CH_3_). MS (EI, 70 eV), *m*/*z* (rel. int.): 150 (6), 132 (45), 117 (100), 105 (12), 92 (36), 91 (86), 78 (23), 77 (16), 65 (14), 51 (10), 45 (24).

Esters **2**, **4**–**10** were prepared from **1**, either as single products (**2**, **8**) or as components of combinatorial mixtures (**4**–**7**, **9**–**10**), using the Steglich esterification procedure [21]. In a typical experiment, a mixture of **1** (152 mg, 1 mmoles, 1 eq.), dicyclohexylcarbodiimide (DCC) (262 mg, 1.3 mmoles, 1.3 eq.), dimethylaminopyridine (DMAP) (13 mg, 0.1 mmoles, 0.1 eq.), and 1.3 mmoles (1.3 eq.) of a carboxylic acid or a mixture of carboxylic acids was stirred in dichloromethane (1.1 mL) for 16 h. Then, 2 mL of petroleum ether was added to the heterogeneous milky mixture, which was subsequently filtered on a pad of celite^®^ and silica gel 40–60 μm (3 g). The celite^®^/silica pad was then rinsed with 15 mL of petroleum ether/diethyl ether 9/1 *v*/*v*, and the filtrate was evaporated to provide the ester or mixture of esters, which was analyzed by GC-MS without further purification.

4-phenylbut-2-yl acetate **2**: colorless oil (81%), RI_DB-1ms_ and RI_DB-Wax_: see Table 1, RI_SLB-5ms_ = 1391. ^1^H NMR (500 MHz, CDCl_3_) δ 7.30–7.24 (m, 2H), 7.20–7.14 (m, 3H), 4.98–4.88 (m, 1H), 2.73–2.55 (m, 2H), 2.05–1.99 (s, 3H), 1.98–1.87 (m, 1H), 1.85–1.74 (m, 1H), 1.25 (d, *J* = 6.3 Hz, 3H); ^13^C NMR (126 MHz, CDCl_3_) δ 170.72 (CO), 141.56 (C), 128.43 (CH), 128.32 (CH), 125.93 (CH), 70.54 (CH), 37.60 (CH_2_), 31.85 (CH_3_), 21.32 (CH_2_), 20.04 (CH_3_). MS (EI, 70 eV), *m*/*z* (rel. int.): 149 (0.72), 132 (39), 131 (8), 118 (8), 117 (100), 115 (9), 105 (6), 91 (45), 77 (7), 65 (9), 43 (36).

4-phenylbut-2-yl 2-methylbutyrate **8**: colorless oil (85%), ca. 1/1 diastereomeric mixture not resolved by GC, but some NMR signals resolved. RI_DB-1ms_ and RI_DB-Wax:_ see Table 1, RI_SLB-5ms_ = 1618. ^1^H NMR (500 MHz, CDCl_3_) δ 7.30–7.24 (m, 2H), 7.20–7.15 (m, 3H), 4.96 (sext., *J* = 6.4 Hz, 1H), 2.73–2.56 (m, 2H), 2.40–2.30 (m, 1H), 1.99–1.88 (m, 1H), 1.85–1.64 (m, 2H), 1.57–1.43 (m, 1H), 1.25 (d, *J* = 6.3 Hz, 3H), 1.16 and 1.16 (2·d, *J* = 7 Hz, 1.5H each), 0.94 and 0.93 (2·t, *J* = 7 Hz, 1.5H each). ^13^C NMR (126 MHz, CDCl_3_) δ 176.48 and 176.44 (2·CO), 141.73 (C), 128.53 (CH), 128.42 (CH), 126.02 (CH), 70.12 and 70.11 (2·CH), 41.53 and 41.42 (2·CH), 37.88 and 37.85 (2·CH_2_), 31.94 (CH_2_), 26.92 and 26.91 (2·CH_2_), 20.18 and 20.12 (2·CH_3_), 16.85 and 16.79 (2·CH_3_), 11.79 and 11.74 (2·CH_3_). MS (EI, 70 eV), *m*/*z* (rel. int.): 133 (6), 132 (56), 131 (10), 118 (11), 117 (100), 92 (6), 91 (66), 85 (6), 77 (5), 65 (9), 57 (22).

4-phenylbut-2-yl propionate **4**: RI_DB-1ms_ and RI_DB-Wax:_ see Table 1, RI_SLB-5ms_ = 1481, MS (EI, 70 eV), *m*/*z* (rel. int.): 149 (0.27), 132 (38), 131 (8), 118 (9), 117 (100), 115 (6), 105 (5), 91 (47), 77 (7), 65 (8), 57 (21).

4-phenylbut-2-yl butyrate **5**: RI_DB-1ms_ and RI_DB-Wax:_ see Table 1, RI_SLB-5ms_ = 1571, MS (EI, 70 eV), *m*/*z* (rel. int.): 148 (0.31), 132 (43), 131 (7), 118 (9), 117 (100), 105 (5), 91 (52), 77 (6), 71 (13), 65 (7), 41 (7).

4-phenylbut-2-yl isobutyrate **6**: RI_DB-1ms_ and RI_DB-Wax:_ see Table 1, RI_SLB-5ms_ = 1523, MS (EI, 70 eV), *m*/*z* (rel. int.): 133 (7), 132 (54), 131 (7), 118 (10), 117 (100), 105 (4), 91 (53), 71 (9), 65 (7).

4-phenylbut-2-yl isovalerate **7**: RI_DB-1ms_ and RI_DB-Wax:_ see Table 1, RI_SLB-5ms_ = 1623, MS (EI, 70 eV), *m*/*z* (rel. int.): 148 (0.29), 133 (9), 132 (61), 131 (8), 118 (9), 117 (100), 91 (48), 85 (9), 65 (6), 57 (14), 41 (7).

4-phenylbut-2-yl benzoate **9**: RI_DB-1ms_ and RI_DB-Wax_: see Table 1, RI_SLB-5ms_ = 1983 MS, (EI, 70 eV), *m*/*z* (rel. int.): 132 (66), 131 (10), 118 (10), 117 (100), 115 (7), 105 (43), 91 (39), 77 (42), 65 (8), 51 (11).

4-phenylbut-2-yl phenylacetate **10**: RI_DB-1ms_ and RI_DB-Wax_: see Table 1, RI_SLB-5ms_ = 2029, MS (EI, 70 eV), *m*/*z* (rel. int.): 133 (4), 132 (44), 118 (4), 117 (40), 105 (3), 92 (8), 91 (100), 89 (3), 77 (3), 65 (12).

## 5. Conclusions

The present study further illustrates the chemical singularity of the Middle Gobi chemotype of *Artemisia rutifolia*, characterized by the occurrence of uncommon phenylbutanoid derivatives in its essential oil. The identification of several previously unreported 4-phenylbut-2-yl esters expands the known structural diversity of natural phenylbutanoids and emphasizes the value of combining targeted fractionation with GC analyses and synthetic reference compounds obtained by combinatorial synthesis for the characterization of minor volatile constituents. Beyond the analytical aspects, the occurrence of these compounds raises interesting questions regarding their biosynthetic origin, since their carbon skeleton does not correspond to the classical phenylpropanoid pathway typically encountered in aromatic plants. Further studies addressing the metabolic origin and geographical distribution of this unusual chemotype will be necessary to better understand its chemotaxonomic significance and potential interest as a new essential oil for industrial production.

## Data Availability

The original contributions presented in this study are included in the article and Supplementary Materials. Further inquiries can be directed to the corresponding author.

## Supplementary Materials

The following supporting information can be downloaded at: https://www.mdpi.com/article/10.3390/molecules31060926/s1.

## Abbreviations

The following abbreviations are used in this manuscript:

GC	Gas Chromatography
NMR	Nuclear Magnetic Resonance
GC-O	Gas Chromatography-Olfactometry
LRI	Linear Retention Index
