# Supplementary material for: 4-Phenylbut-2-yl Esters from the Essential Oil of Artemisia rutifolia from Mongolia
Source: Molecules. 2026 Mar 11;31(6):926. doi: 10.3390/molecules31060926 (PMC13029260; doi:10.3390/molecules31060926)
Supplement: Supplementary file 1 [file molecules-31-00926-s001.zip › molecules-4184473-supplementary.pdf]

# Supplementary Material

# 4-Phenylbut-2-yl Esters from the Essential Oil of *Artemisia rutifolia* from Mongolia

**Elisa Irrera [1,2,†], Yea Jee Ahn [1,†], Shatar Sandui [3], Altantsetseg Shatar [3] and Nicolas Baldovini [1,*]**

[1] Institut de Chimie de Nice, UMR 7272, Université Côte d'Azur, Parc Valrose, 06108 Nice, France; elisa.irrera@studenti.unime.it (E.I.); yeajeeahn@gmail.com (Y.J.A.)
[2] Messina Institute of Technology c/o Department of Chemical, Biological, Pharmaceutical and Environmental Sciences, University of Messina, Viale G. Palatucci 13, 98168 Messina, Italy
[3] Institute of Chemistry and Chemical Technology, Mongolian Academy of Sciences (MAS), Ulaan-Baatar 211051, Mongolia; altaa12000@yahoo.com (A.S.)
* Correspondence: nicolas.baldovini@unice.fr; Tel.: +33-492076132
† These authors contributed equally to this work.

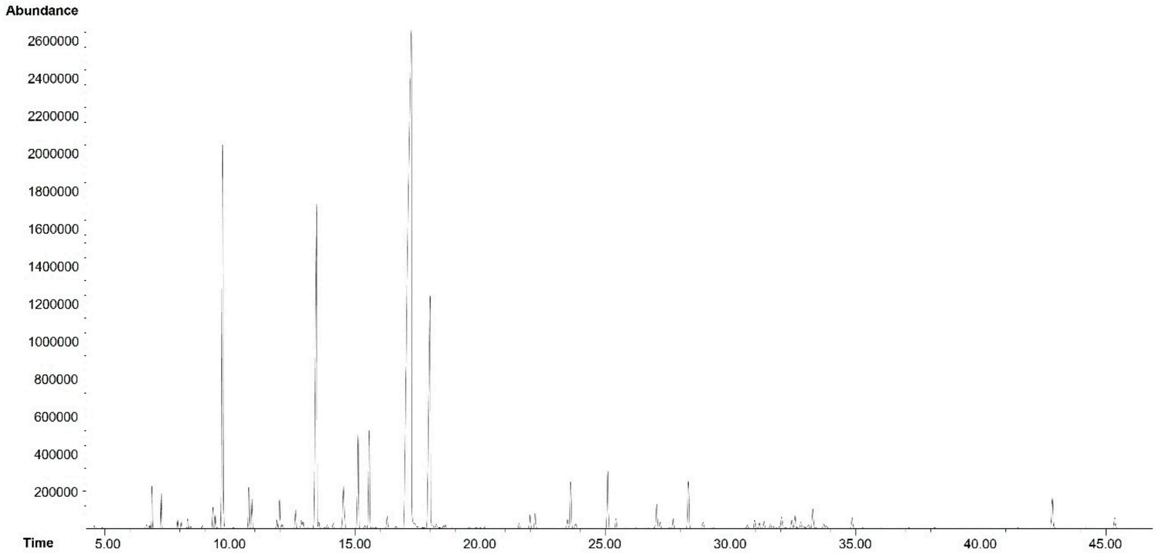


**Figure S1.** Chromatogram of *A. rutifolia* essential oil analyzed by DB-1ms columns.

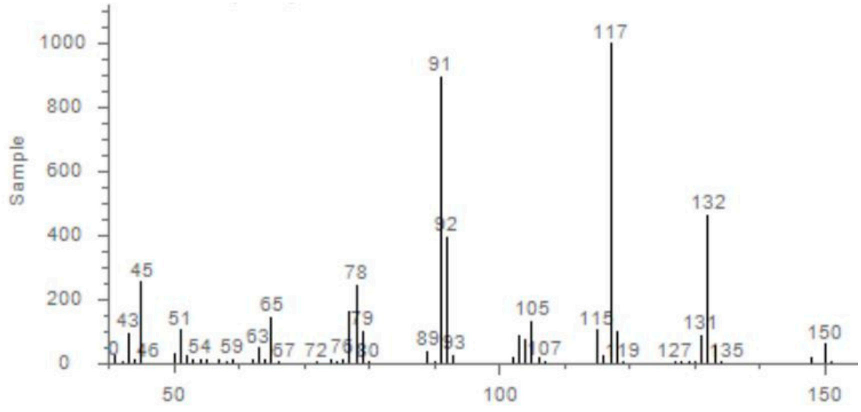


**Figure S2.** 4-phenylbutan-2-ol (1) MS spectrum.

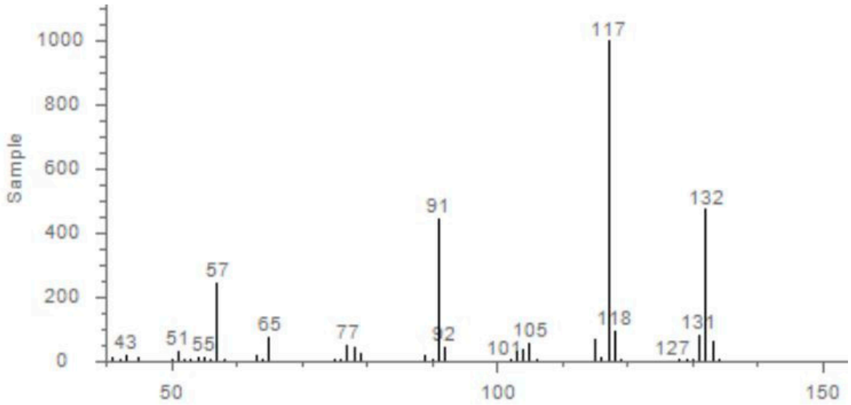


**Figure S3.** 4-phenylbut-2-yl acetate (2) MS spectrum.

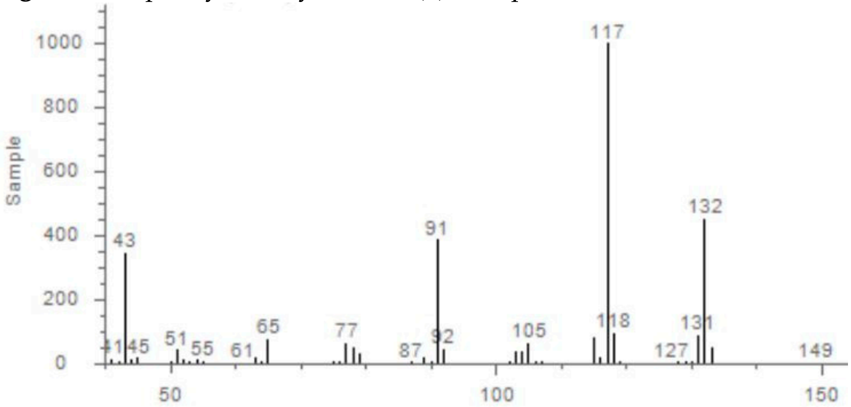


**Figure S4.** 4-phenylbut-2-yl propionate (4) MS spectrum.

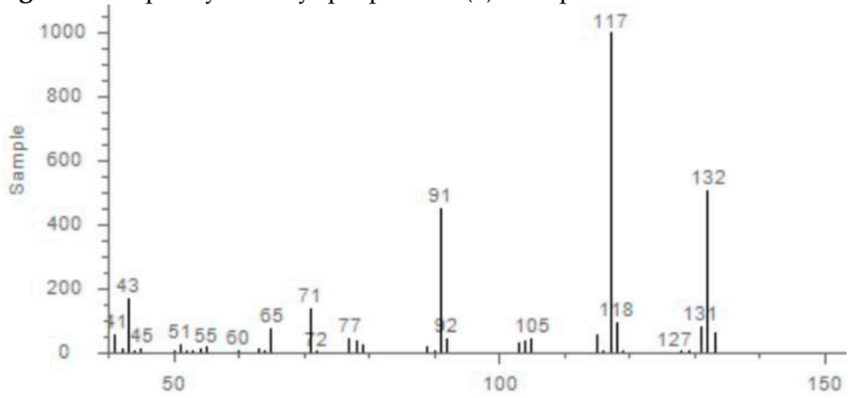


**Figure S5.** 4-phenylbut-2-yl butyrate (5) MS spectrum.

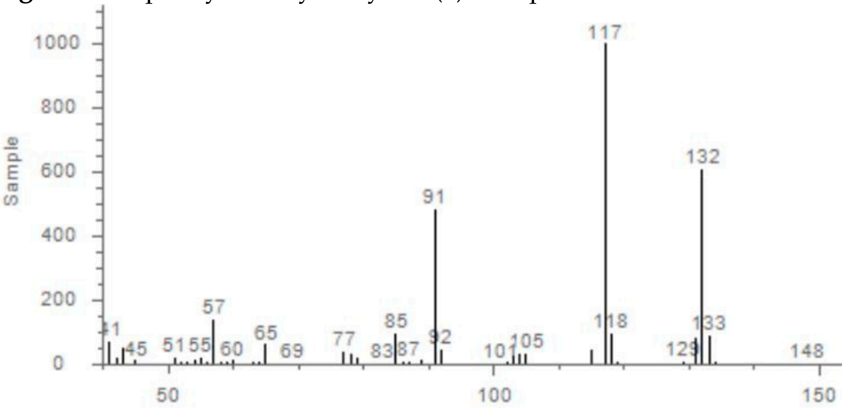


**Figure S6.** 4-phenylbut-2-yl isovalerate (7) MS spectrum.

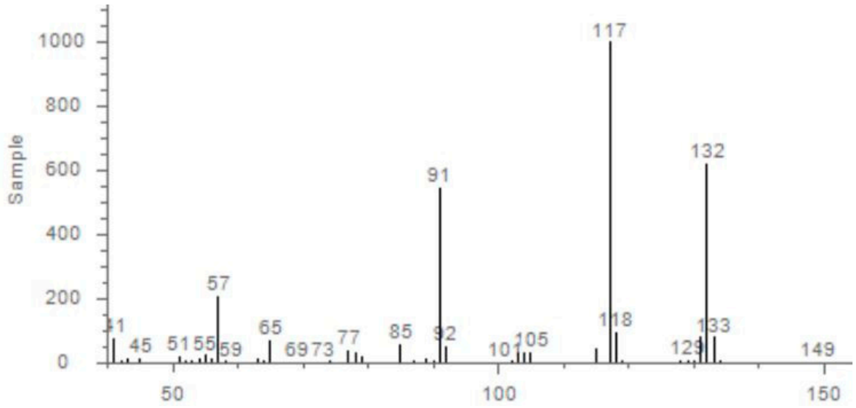


**Figure S7.** 4-phenylbut-2-yl 2-methylbutyrate (8) MS spectrum.

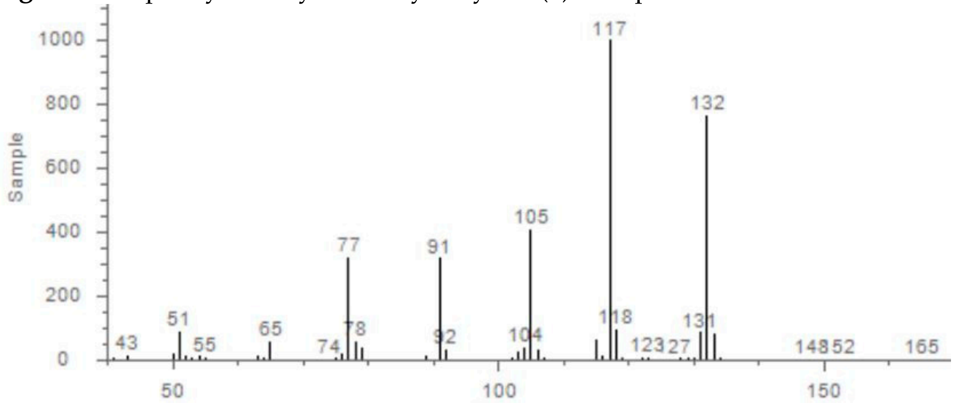


**Figure S8.** 4-phenylbut-2-yl benzoate (9) MS spectrum.

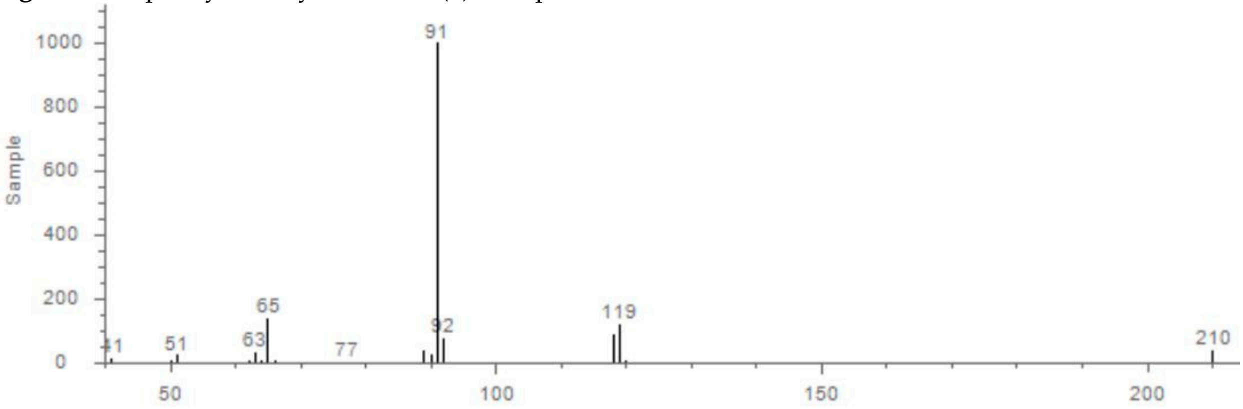


**Figure S9.** 4-phenylbut-2-yl phenylacetate (10) MS spectrum.

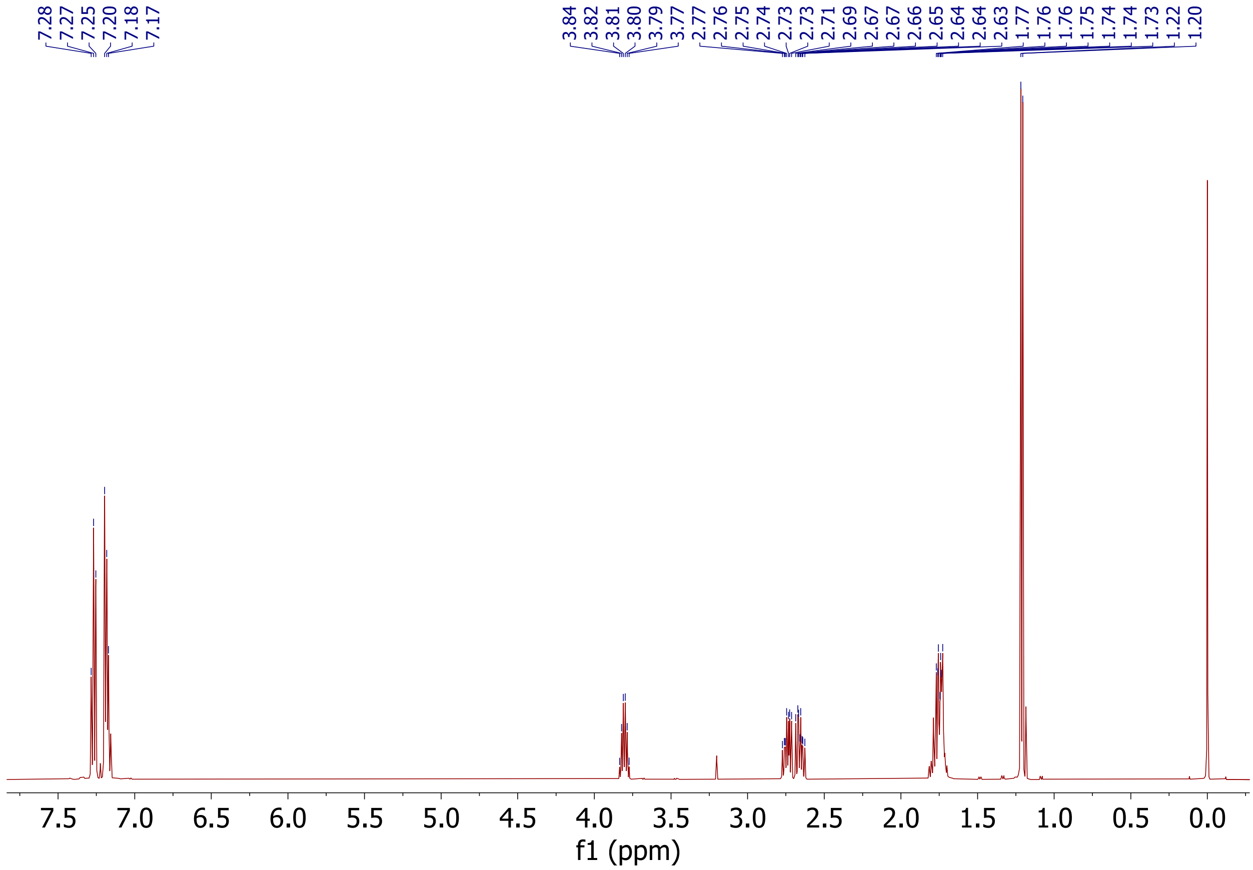


**Figure S10.** 4-phenylbutan-2-ol $^{1}$H NMR spectrum.

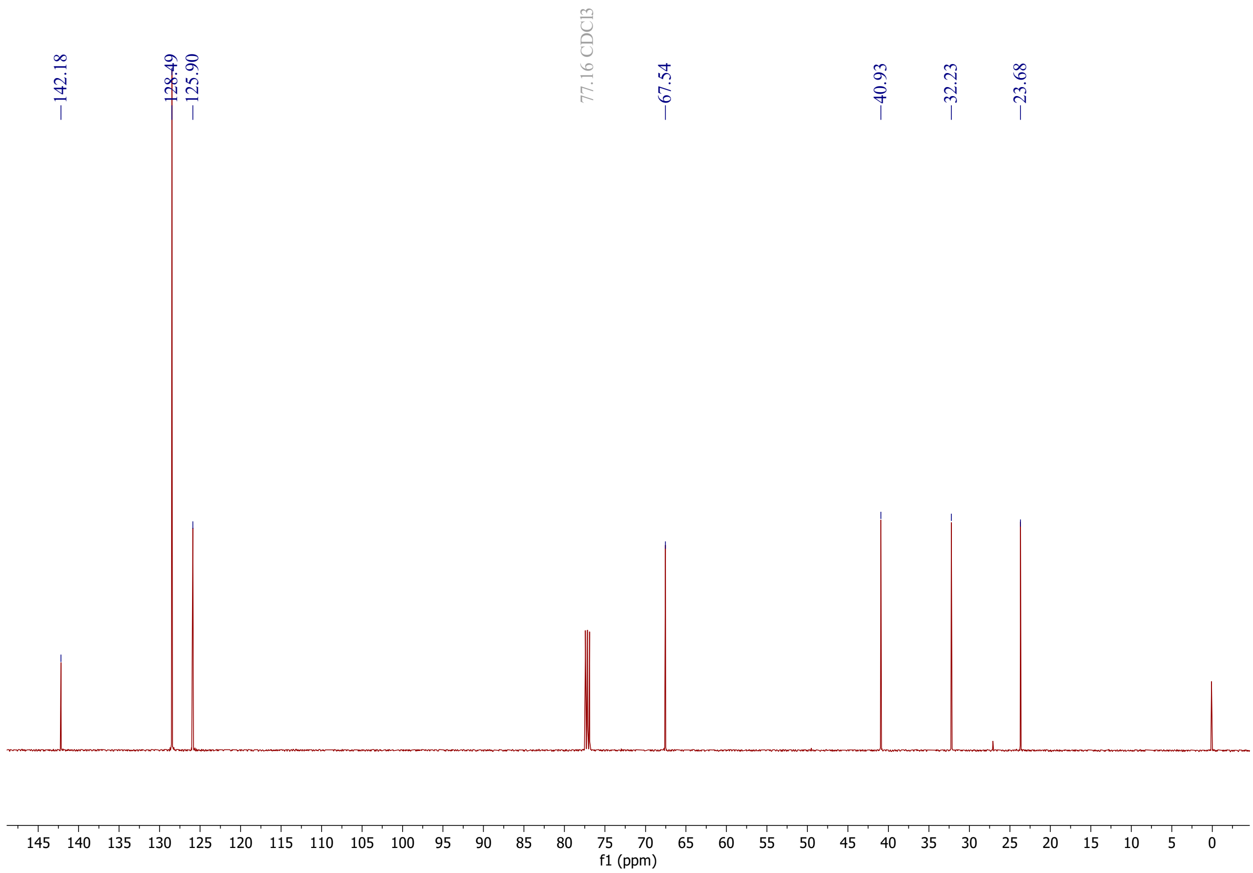


**Figure S11.** 4-phenylbutan-2-ol $^{13}$C NMR spectrum.

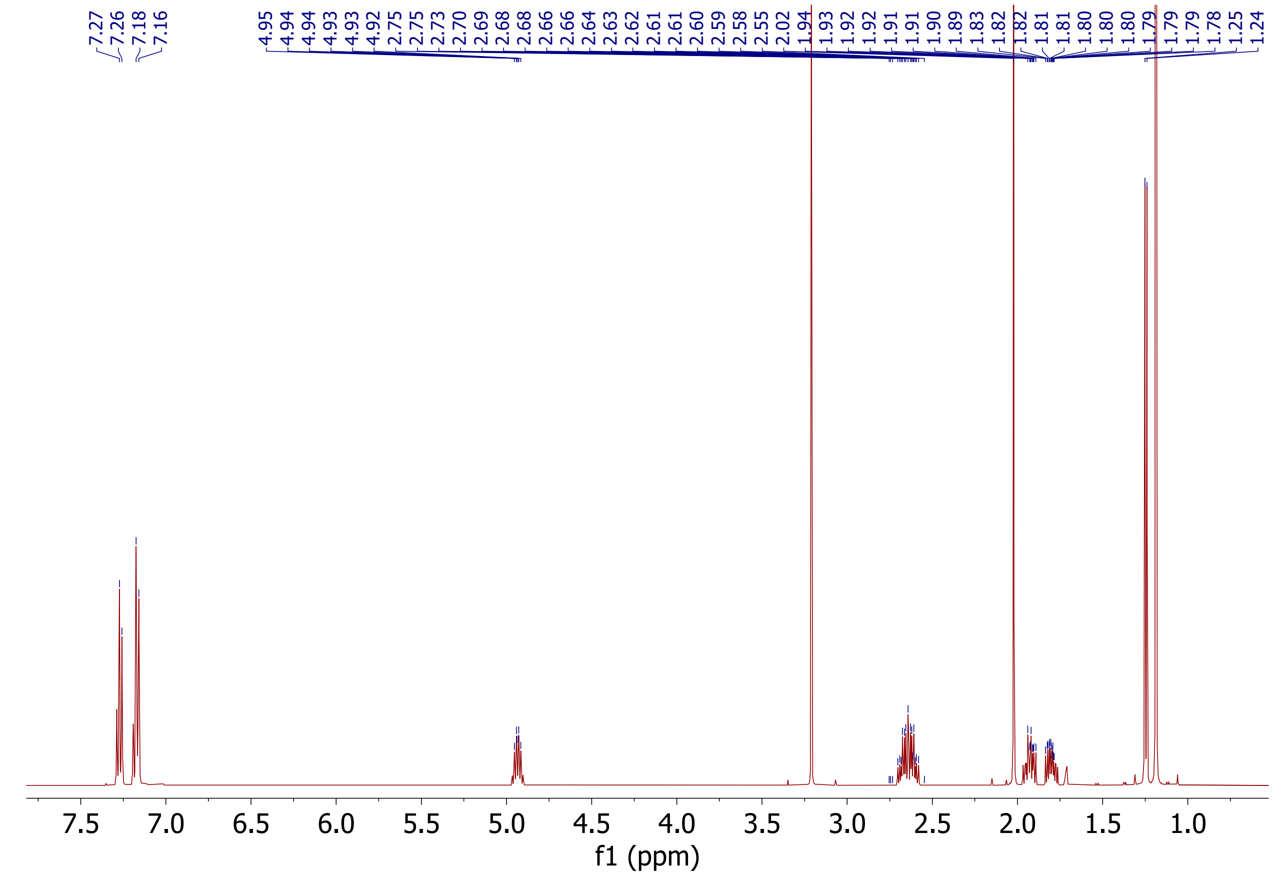


**Figure S12.** 4-phenylbut-2-yl acetate $^1$H NMR spectrum.

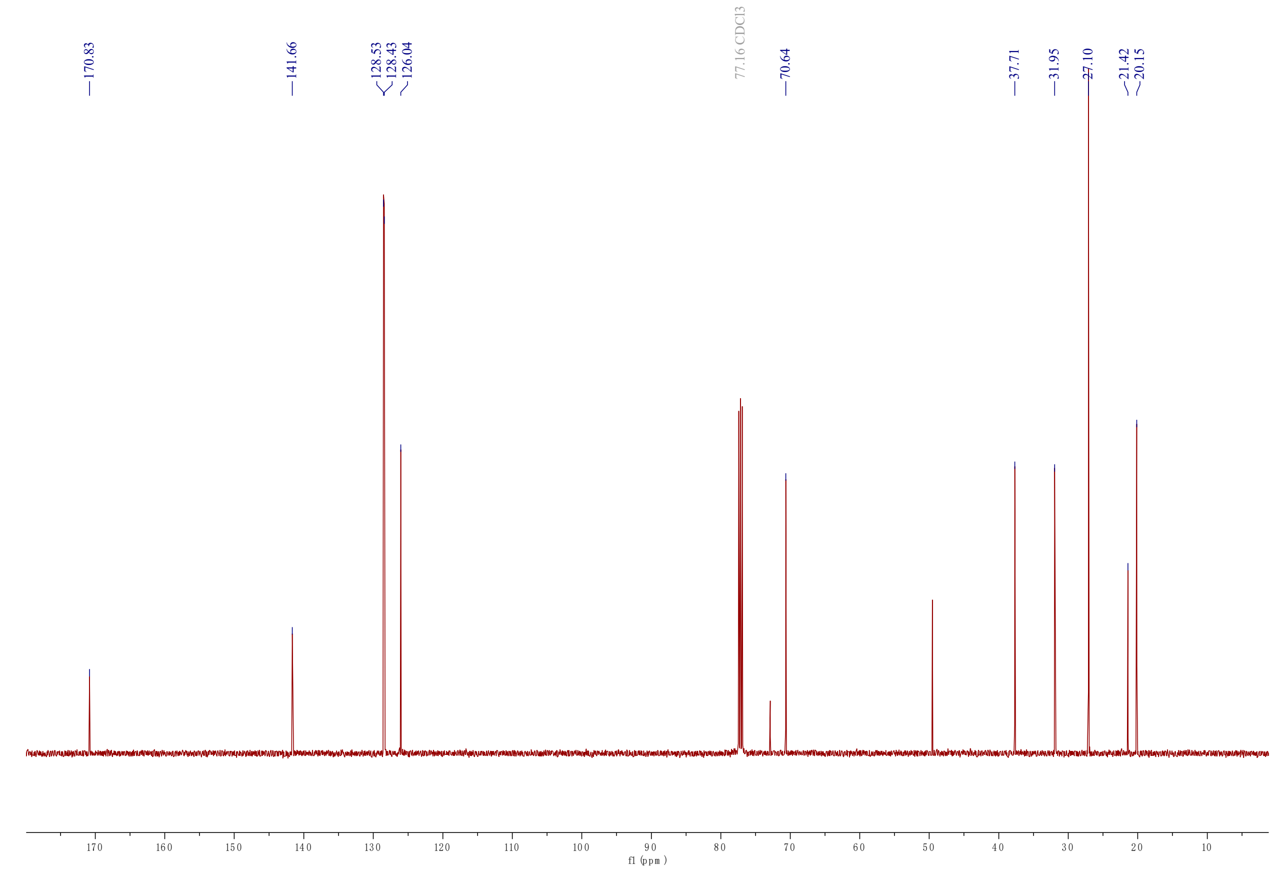


**Figure S13.** 4-phenylbut-2-yl acetate $^{13}$C NMR spectrum.

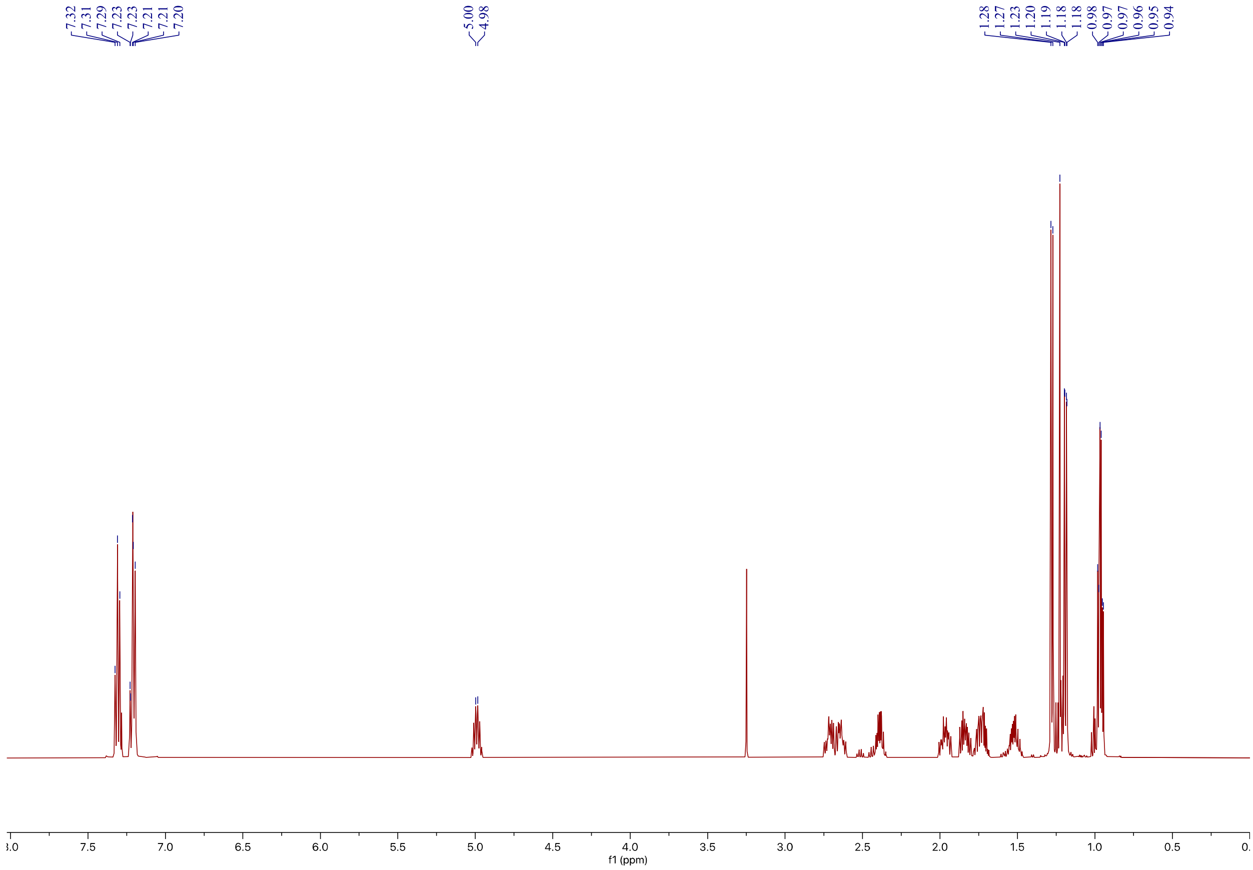


**Figure S14.** 4-phenylbut-2-yl 2-methylbutyrate $^{1}$H NMR spectrum.

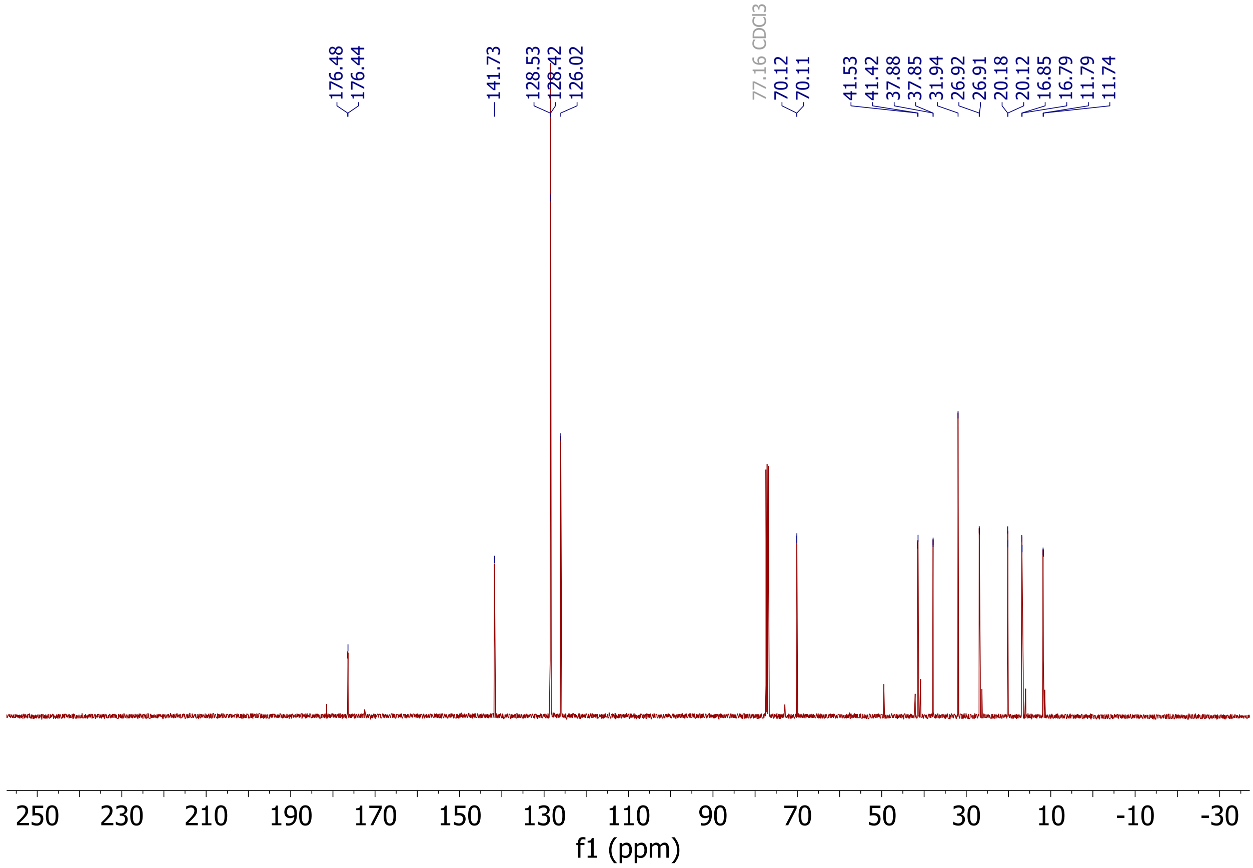


**Figure S15.** 4-phenylbut-2-yl 2-methylbutyrate $^{13}$C NMR spectrum.
